# Multiple Novel Clades of Anopheline Mosquitoes Caught Outdoors in Northern Zambia

**DOI:** 10.3389/fitd.2021.780664

**Published:** 2021-12-09

**Authors:** Christine M. Jones, Ilinca I. Ciubotariu, Mbanga Muleba, James Lupiya, David Mbewe, Limonty Simubali, Twig Mudenda, Mary E. Gebhardt, Giovanna Carpi, Ashley N. Malcolm, Kyle J. Kosinski, Ana L. Romero-Weaver, Jennifer C. Stevenson, Yoosook Lee, Douglas E. Norris, Southern Central Africa International Centers of Excellence for Malaria Research

**Affiliations:** 1 The W. Harry Feinstone Department of Molecular Microbiology and Immunology, The Johns Hopkins Malaria Research Institute, Johns Hopkins Bloomberg School of Public Health, Baltimore, MD, United States,; 2 Department of Biological Sciences, Purdue University, West Lafayette, IN, United States,; 3 Tropical Diseases Research Centre, Ndola, Zambia,; 4 Macha Research Trust, Choma, Zambia,; 5 Florida Medical Entomology Laboratory, Department of Entomology and Nematology, Institute of Food and Agricultural Sciences, University of Florida, Vero Beach, FL, United States

**Keywords:** mosquito, phylogenetics, malaria, residual transmission, *Anopheles*, Zambia

## Abstract

Residual vector populations that do not come in contact with the most frequently utilized indoor-directed interventions present major challenges to global malaria eradication. Many of these residual populations are mosquito species about which little is known. As part of a study to assess the threat of outdoor exposure to malaria mosquitoes within the Southern and Central Africa International Centers of Excellence for Malaria Research, foraging female anophelines were collected outside households in Nchelenge District, northern Zambia. These anophelines proved to be more diverse than had previously been reported in the area. In order to further characterize the anopheline species, sequencing and phylogenetic approaches were utilized. Anopheline mosquitoes were collected from outdoor light traps, morphologically identified, and sent to Johns Hopkins Bloomberg School of Public Health for sequencing. Sanger sequencing from 115 field-derived samples yielded mitochondrial COI sequences, which were aligned with a homologous 488 bp gene segment from known anophelines (n = 140) retrieved from NCBI. Nuclear ITS2 sequences (n = 57) for at least one individual from each unique COI clade were generated and compared against NCBI’s nucleotide BLAST database to provide additional evidence for taxonomical identity and structure. Molecular and morphological data were combined for assignment of species or higher taxonomy. Twelve phylogenetic groups were characterized from the COI and ITS2 sequence data, including the primary vector species *Anopheles funestus* s.s. and *An. gambiae* s.s. An unexpectedly large proportion of the field collections were identified as *An. coustani* and *An*. sp. *6*. Six phylogenetic groups remain unidentified to species-level. Outdoor collections of anopheline mosquitoes in areas frequented by people in Nchelenge, northern Zambia, proved to be extremely diverse. Morphological misidentification and underrepresentation of some anopheline species in sequence databases confound efforts to confirm identity of potential malaria vector species. The large number of unidentified anophelines could compromise the malaria vector surveillance and malaria control efforts not only in northern Zambia but other places where surveillance and control are focused on indoor-foraging and resting anophelines. Therefore, it is critical to continue development of methodologies that allow better identification of these populations and revisiting and cleaning current genomic databases.

## INTRODUCTION

Human malaria is transmitted by species of mosquitoes in the genus *Anopheles*. There are approximately 450 recognized species of anopheline mosquitoes worldwide, which are placed into six main subgenera: *Anopheles*, *Cellia*, *Kerteszia*, *Lophopodomyia*, *Nyssorhynchus*, and *Stethomyia*. The largest of these subgenera by far are *Anopheles* (183 species) and *Cellia* (224 species) (1). *Cellia* has an old-world distribution, *Anopheles* is cosmopolitan, and the remaining subgenera are neotropical in distribution. Despite this diversity, there are fewer than 50 species within the entire *Anopheles* genus that are classically considered important to maintaining human malaria transmission (2).

Subgenera of anophelines can be further divided into smaller taxonomic units, including Sections, Series, Groups, and species complexes. Members of species complexes are morphologically difficult to distinguish, so a combination of morphological, behavioral/ecological, and molecular approaches must be used to identify species (3). Species-level identification is important because even at the level of highly-related species within a complex, behavior, ecology, vector competence and susceptibility to vector control can be highly variable (4–11). Members of the *An. funestus* sensu lato (s.l.) group, for example, differ in terms of host preference, foraging behavior, insecticide resistance, and ecological niche (12–14). For well-studied systems like the *Anopheles gambiae* complex, it has been repeatedly shown the importance of good genetic markers for any association studies investigating insecticide resistance (9, 15) or vector competence (11, 16).

In pre-elimination settings where the principal malaria vectors have been largely reduced, persistent malaria transmission has been frustratingly difficult to control. In these malaria endemic regions, vector surveillance has been challenging partly due to limited resources for molecular and genetic species verification for understudied, undescribed or morphologically cryptic anophelines. To date, most phylogenetic studies have focused on classically-recognized malaria vectors (17). Although morphology remains the primary and most cost-effective method for anopheline identification, newly-discovered and largely undescribed anopheline species are increasingly recognized as important to malaria transmission (18–23), and the scarcity of genetic data for development of molecular diagnostics for a wider range of anopheline species significantly hinder rapid confirmation of potential vectors of malaria transmission (24).

**FIGURE 1 |:**
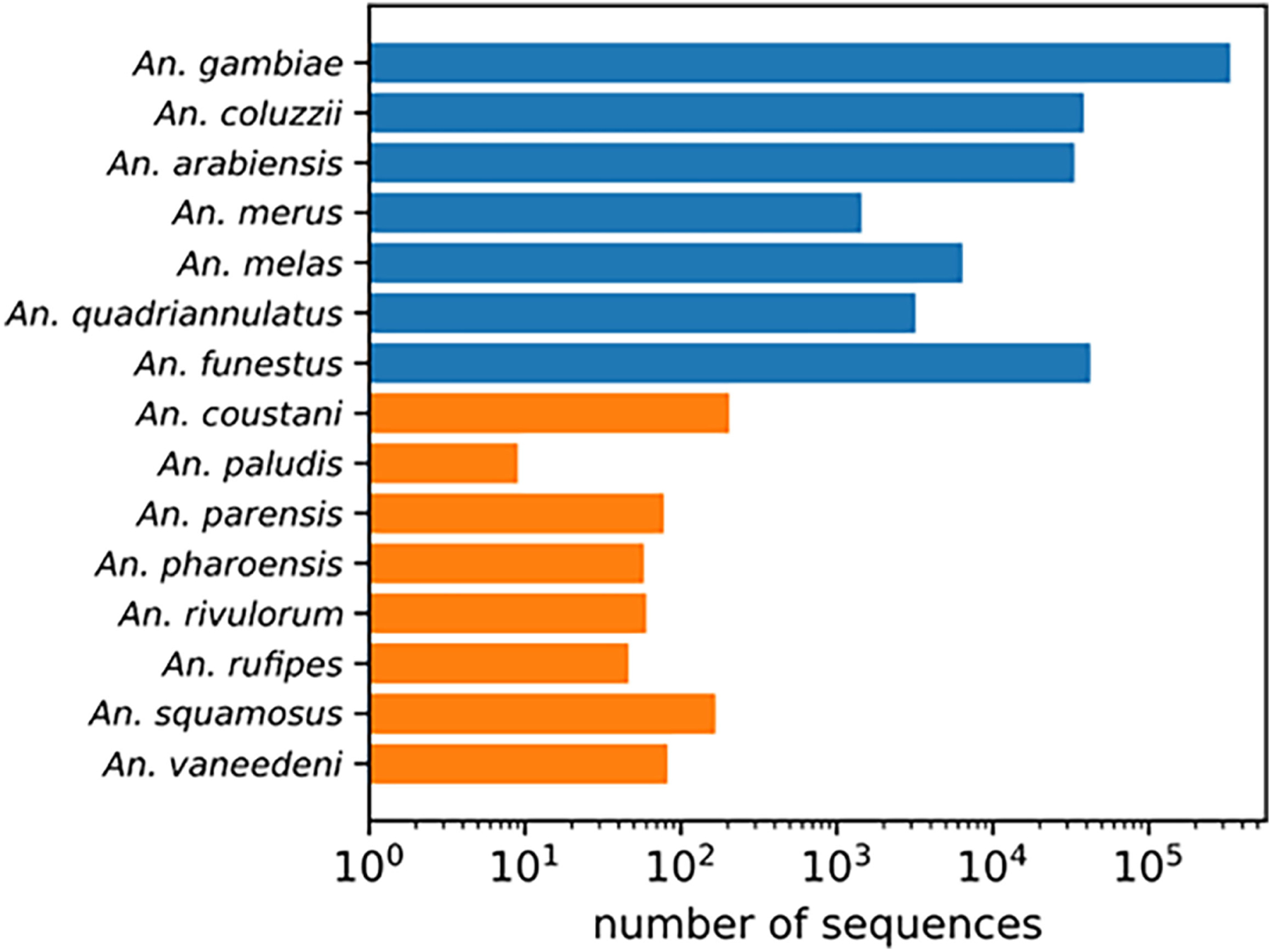
NCBI Nucleotide database search results for each species name. Well-studied *An. gambiae* complex sibling species and *An. funestus* s.s. in blue. Understudied species in orange.

When diverse specimens from field collections remain unidentified using routine morphological and polymerase chain reaction (PCR)-based methods, sequence comparison to National Center for Biotechnology Information (NCBI) non-redundant nucleotide database using the Basic Local Alignment Search Tool (BLAST) may be used (25). This method has limited power for identifying understudied vector species because of the paucity of well-documented reference sequences for neglected anopheline species. For example, *Anopheles gambiae* has over 300,000 sequences in the NCBI Nucleotide database. Other well studied members of the *Anopheles gambiae* complex and *An. funestus* have just over 1000 entries in the NCBI Nucleotide database. In contrast, understudied species have at most 200 entries and most of them have under 80 entries (Figure 1). Due to recent changes some species designations (3) these could be misidentified. Unverified specimens, due to discrepancies in feature annotations, could also be excluded from a BLAST search. As this is much more common for understudied species, a BLAST search may have limited utility as a method of species identification, depending on genomic target.

The mitochondrial cytochrome oxidase c subunit I (COI) gene is often targeted for species-level identification as a so-called ‘barcode’ for many taxa, including many vector insects and anophelines (26–29). The COI gene is targeted because efforts to catalogue global species diversity using the Barcode of Life (BOL) have led to many sequences being available for this locus (30), and the balance of sequence conservation and polymorphism in COI allow for comparison at the level of closely related species, such as within the complicated species complexes common to anophelines. However, reliance on mitochondrial loci for species relatedness could be problematic for mosquitoes because researchers have demonstrated the lack of species-specific markers in the *An. gambiae* complex (31) or different gene genealogies between mitochondrial and nuclear markers in other mosquito species such as *Ae. aegypti* (32). For this reason, the internal transcribed spacer 2 (ITS2) ribosomal region could be used an alternative target in the nuclear genome for species identification. In the *Anopheles gambiae* complex, ITS2 experiences a higher rate of divergence than COI and has better discriminating power for phylogenetic relationships at the level of species complexes (31, 33). The use of these complementary targets also allows for a fuller picture of phylogenetic relationships, with COI representing mitochondrial/maternal inheritance and ITS2 reflecting nuclear inheritance (24).

A few recent studies have combined morphological data and sequencing of COI and ITS2 to illuminate previously-unrecognized diversity of anophelines in southern and east Africa (20, 34, 35). In eastern Zambia, molecular examination found a large number of species and species groups which had been misidentified solely using morphological identification (34). Malaria parasites were detected in many of these specimens. In numerous instances, these anophelines did not have a DNA sequence match in publicly available databases (34). This was not only observed in Zambia. Studies from western Kenya also demonstrated high species diversity of indoor and outdoor collections of anophelines, with infected specimens found in anopheline species with no corresponding published molecular sequences (20). From the outdoor collections, one species, designated *An*. sp. *1*, dominated collections with an infection rate similar to that seen for *An. funestus* (20). The presence of such cryptic but significant malaria vectors escaping typical malaria entomological surveillance seriously compromises the assessment and control of malaria.

Indoor-based collections have been conducted extensively in Nchelenge district, northern Zambia. From these collections, *An. funestus* s.s. is considered the primary vector both as a result of a high malaria parasite infection rate and its occurrence in substantially higher numbers than other anophelines. *Anopheles gambiae* is a secondary contributor to transmission in this region (36–38). *Anopheles coustani* and *An. leesoni*, which are not traditionally considered malaria vector species in Zambia, are found in small numbers indoors, and only a handful of other anopheline species have been identified in the area (36, 37, 39). To date, collections have been almost exclusively focused indoors and indoor-focused interventions, such as widespread use of long-lasting insecticide treated nets (LLINs) and indoor residual spraying (IRS), have had minimal impact in reducing malaria in Nchelenge (40–42).

Based on these facts, we hypothesized that there could be unrecognized vectors contributing to malaria transmission that are missed by solely relying on indoor surveillance and that are not targeted by indoor-directed vector control efforts. In order to capture other anophelines, we utilized Centers for Disease Control light traps (CDC LTs) in outdoor locations in Nchelenge District. We sequenced COI and ITS2 segments of collected specimens and searched the NCBI Nucleotide database to find matching species. Here, we report our findings on species diversity, species identification success rates, and implications on future research, surveillance, and control of malaria vectors in Zambia and Africa.

## MATERIALS AND METHODS

### Sample Collection

**FIGURE 2 |:**
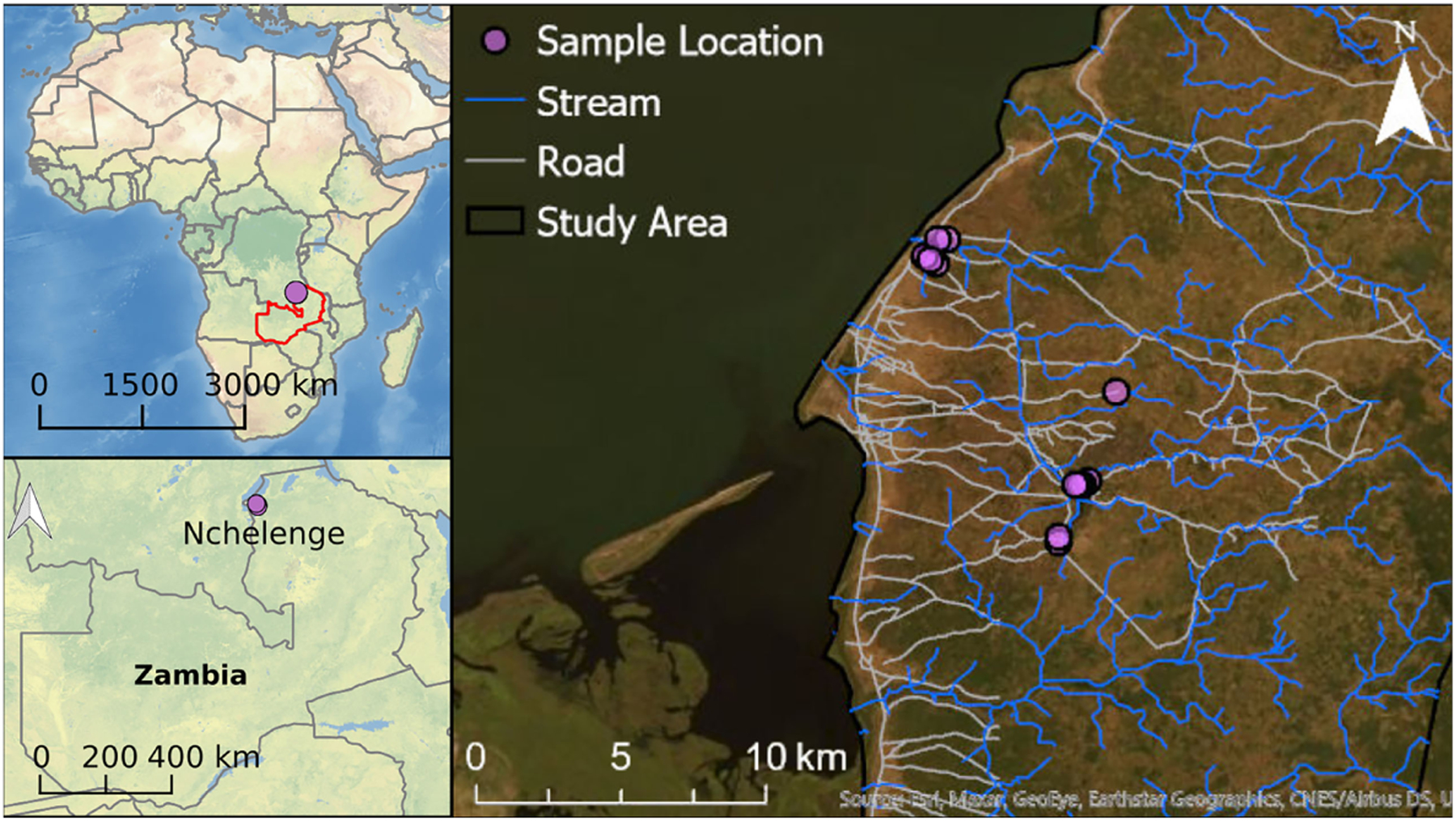
Collection site map. Each purple dot represents a household in Nchelenge. The public domain map, CleanTopo2 (43) was used for the base map on left two panels. World imagery from ArcGIS® software by Esri was used for the base map for the right panel.

*Anopheles* mosquitoes were collected from outdoor locations in Nchelenge District in northern Zambia. Nchelenge shares a border with the Democratic Republic of the Congo that bisects Lake Mweru. It is a marshy region lying ~800 meters above sea level. There are three seasons: a rainy season from November to May, a cool dry season from May to August, and a hot dry season from August to November. *Anopheles funestus* and *An. gambiae* are considered the primary vectors in the area, although *An. funestus* contributes more to transmission in Nchelenge due to its much greater abundance and infection rates (37, 39). The *An. funestus* population peaks during the dry season when *An. gambiae* numbers are at their lowest (37). Sample collections for this study were conducted in August 2016 during the dry season, through which high malaria transmission is maintained. CDC LTs were set in a total of thirteen households in two different locations in Nchelenge, either within a kilometer from the lake or in a village more than 7 kilometers inland (Figure 2). Traps were set outside overnight adjacent to areas where people gathered in the evening, close to outdoor washing areas/latrines, and near animal pens. Traps were rotated through households for nine nights. Due to samples lost during shipping, the collections here represent collections from a total of 74 trap nights.

### DNA Extraction and Species Identification

Mosquitoes were morphologically identified to species using standard keys by trained personnel (12, 13). Anopheline mosquitoes were placed individually into labelled 0.6 mL microcentrifuge tubes containing silica gel desiccant and cotton wool and stored at room temperature. Abdomens and heads/thoraces were split and placed into two separate tubes. DNA was extracted from abdomen tissue of each mosquito using a modified salt extraction protocol as previously described (36, 39, 44).

For anopheline molecular species identification, a series of PCR assays was conducted and products visualized by electrophoresis on 2% agarose gels. For specimens morphologically identified as members of the *An. funestus* or *An. gambiae* complex, respective species-complex-specific PCR diagnostics were run (45, 46). For specimens that did not amplify following species-complex-specific PCR diagnostics for *An. funestus* or *An. gambiae* species groups or for which morphological identification indicated they were not of these two major vector groups, a more general differential PCR based on the ITS2 region of rDNA was used (26, 35, 47). For those samples that either did not amplify using the ITS2 assay, or that gave an ambiguous fragment size, a COI-based BOL (cytochrome oxidase I - Barcode of Life) PCR protocol was used to amplify a ~500 bp long fragment followed by Sanger sequencing (34). When samples failed to amplify, pellets from the abdomen DNA extraction that had been saved and stored at −20°C were re-extracted using the Qiagen DNeasy Blood and Tissue Kit (Qiagen, Hilden, Germany), and the product was again subjected to the COI PCR assays.

### Sequencing and Phylogenetic Analysis

Samples that amplified following the COI-based BOL PCR were purified using the QIAquick PCR Purification Kit (Qiagen, Hilden, Germany). COI BOL amplicons were sequenced at the Johns Hopkins Medical Institutions (JHMI) Synthesis and Sequencing Facility using the LCO1490 (5’-GGT CAA CAA ATC ATA AAG ATA TTG G-3’) and HCO2198 (5’-TAA ACT TCA GGG TGA CCA AAA AAT CA-3’) primers described by Hebert et al. (26). Forward and reverse sequences were trimmed to remove ends with low quality and then high-quality trimmed sequences were aligned to generate a single consensus sequences using Geneious v11.1.5 (48). Corresponding sequences of the COI from known anopheline species, as well as several taxonomically unassigned species (e.g. “*An*. sp. *1*”), were downloaded from the NCBI database (n = 140) to represent a spectrum of taxa in the genus *Anopheles* as well as several sequences from sister genera for analysis as outgroups (Supplementary Table S1). All sequences (n = 256) were trimmed to a final length of 488 bp and aligned in Geneious v11.1.5 (48). Identical sequences were collapsed to a single unique sequence for analysis using FaBox, resulting in a final 196 unique sequences (49). Phylogenetic trees were built using the Jukes-Cantor Genetic Distance method (50) and the Neighbor-Joining tree build method (51) implemented in Geneious (48). Two hundred replicates were used to calculate bootstrap values. Nodes with low support (lower than 75% support threshold) were collapsed into polytomies, and an *Ae. aegypti* sequence or most distant *Anopheles* were used as outgroups.

**FIGURE 3 |:**
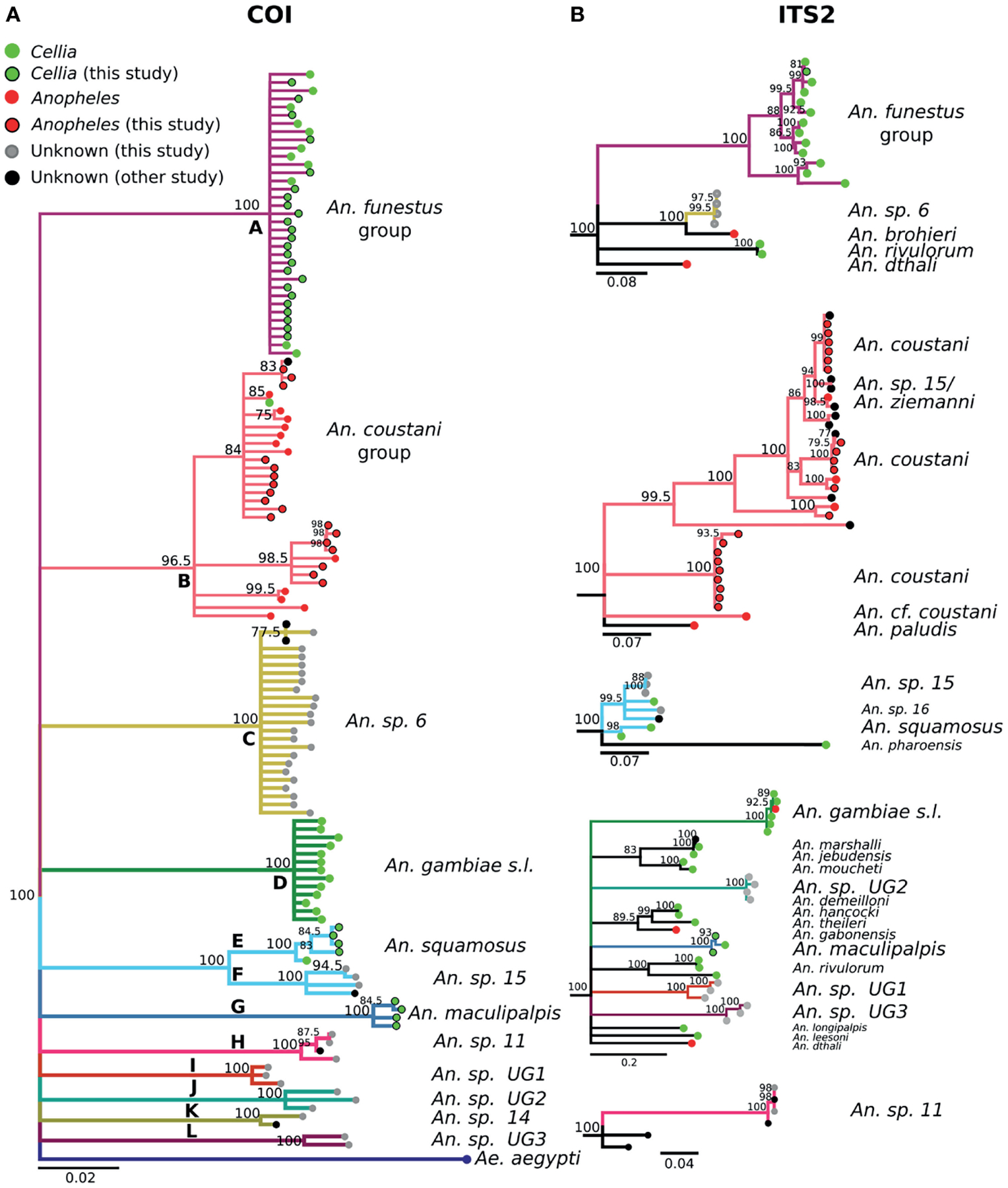
**(A)** Phylogenetic tree based on COI sequences. **(B)** Tree based on ITS2 sequences. Trees include both well-defined sequences from this study (green or red node termini outlined in black) and from NCBI (green or red node termini without black outline), as well as sequences without a proper species identification (gray from this study and black from NCBI).

To validate results from COI BOL sequencing, representative specimens of each phylogenetic group from the COI tree (Figure 3A) were sequenced using the internal transcribed spacer region (ITS2) in the nuclear genome. ITS2 amplicons were purified and sent for sequencing using the forward and reverse ITS2A (5’- TGT GAA CTG CAG GAC ACA T -3’) and ITS2B (5’- TAT GCT TAA ATT CAG GGG GT -3’) primers (46). Individual forward and reverse sequences were trimmed to remove low quality ends and then the resulting trimmed high-quality sequences were aligned in a pairwise fashion to generate single consensus sequences. In the few cases where either the forward or reverse sequence of a samples failed, the single high-quality trimmed read was used instead for further analyses. Final ITS2 sequences were compared using BLASTN against the NCBI non-redundant nucleotide database. Hits with a high percentage of query coverage (>70%), a high percentage sequence identity (>80%), and/or E-value < 1×10^−21^ were considered good hits.

### Annotation and Data Availability

COI sequences generated in this study are available in GenBank with the following accession numbers: MK016543–MK016657. ITS2 sequences accession numbers are: MK592014–MK592096.

### Map Generation

Maps of anopheline species composition at geolocated study households were created with QGIS version × QGIS version 3.18 (https://www.qgis.org/). CleanTOPO2 (43) World imagery from ArcGIS^®^ software by Esri was used for the base map.

## RESULTS

A total of 790 female anophelines were molecularly processed for this study, generating 115 representative COI BOL sequences from morphological groups to assist with species verification. For 43/790 (5.4%) samples, which repeatedly failed to amplify for any PCR attempted, genetic data could not be generated, and thus were excluded from subsequent analyses. Of those 43 samples, 25 (58.1%) were morphologically identified as *An. funestus*, 7 (16.3%) were *An. coustani*, 7 (16.3%) were inconclusive, 3 (7.0%) were *An. squamosus/cydippis*, and 1 (2.3%) was *An. gambiae*. The majority of the species-validated specimens (as determined through a combination of morphological and molecular analyses) were *An. funestus* s.s. (644/747, 86.2%), with only a few *An. gambiae* s.s. (14/747, 1.9%), and an unexpectedly high diversity of additional species (89/747, 11.9%, Table 1).

### Phylogenetic Analyses

The 488 bp multi-alignment for the COI BOL included 132 unique haplotypes and 98 ITS2 sequences were used to construct phylogenetic trees (Figure 3). *Anopheles funestus* morphological identifications were confirmed by molecular and sequence analysis and these samples fell in a single well-supported clade with NCBI sequences from *An. funestus* group (Figure 3, clade A). Two sequenced *An. gambiae* samples fell into a well-supported clade with sequences representing the *An. gambiae* complex (data not shown). The phylogenetic tree revealed approximately 10 additional clades of sequenced study specimens outside of *An. funestus* and *An. gambiae* (Table 1 and Figure 3).

Some species groups and complexes clustered into well-supported clades using COI, which is consistent with widespread use of COI as a good discriminator at the approximate level of species groups (26, 30, 31). However, some sequences were clustered in exclusive clades without any recognized species included in them (clades H-L in Figure 3). When possible, we assigned species identifiers from corresponding closely-matched and previously identified species. A total of 12 clades were identified from our collection based on the COI tree (Figure 3A). There were 3 clades, namely I, J, and L, with no match in NCBI. We named these clades as Unknown Group 1 (UG1), UG2, and UG3, respectively. ITS2 sequences were too divergent to construct a single phylogenetic tree with a common root (Figure 3B). We summarized the relationship between morphological identification and sequence-based identification in Table 1.

Species groups *An. funestus, An. coustani*, and *An. gambiae* grouped together independently based on ITS2 sequences (Figure 3B). *An*. sp. *6* sample were distantly related to *An. funestus* group, *An. brohieri*, and *An. rivulorum* (Figure 3B). The *An. coustani* group formed multiple clades with other study samples recognized as *An*. sp. *15* or *An. ziemanni* (Figure 3B), suggesting further studies are needed to resolve members of this species complex. *An. squamosus* and *An*. sp. *15/16* formed distinct but closely related clades (Figures 3A, B). The COI sequence similarity with *An*. sp. *15/16* was over 98% and ITS2 sequence similarity with *An*. sp. *15/16* over 92% (Table 1). The COI tree was not able to resolve the species relationship with clades including *An*. sp. 11 and 14 as well as the UG1-3 groups (Figure 3A), while ITS2 sequences revealed potentially distant relationships (ITS2 sequence similarity between 70–80%) to *An. demeilloni, An. marshalli*, and *An. hancocki* (Table 1 and Figure 3B).

### Subgenus Cellia

**FIGURE 4 |:**
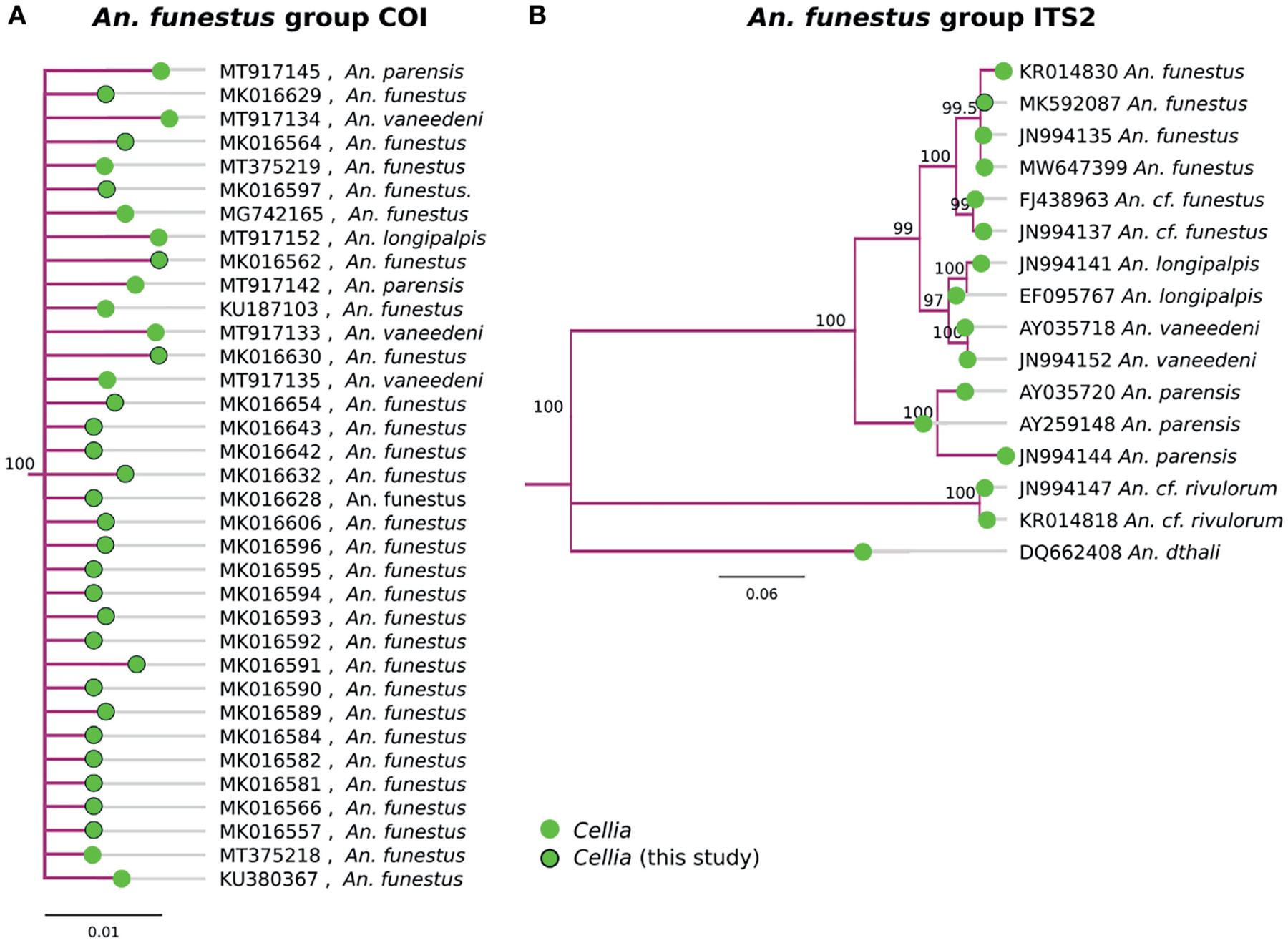
**(A)** Phylogenetic tree based on COI sequences of *An. funestus* group samples. **(B)** Tree based on ITS2 sequences. Trees include both well-defined sequences from this study (green node termini outlined in black) and from NCBI (green node termini without black outline).

Multiple species in the *An. funestus* group, *An. parensis, An. vaneedeni, An. longipalpis*, and *An. funestus* s.s., formed a monophyletic clade based on COI sequences (Figure 4A). ITS2 sequences were better at resolving different member species within the *An. funestus* group and all our samples fall within *An. funestus* s.s. (Figure 3B). Sequence similarity of our *An. funestus* s.s. sample was 85–87% with *An. longipalpis*, 85–86% with *An. vaneedeni*, and 72–75% with *An. parensis*.

**FIGURE 5 |:**
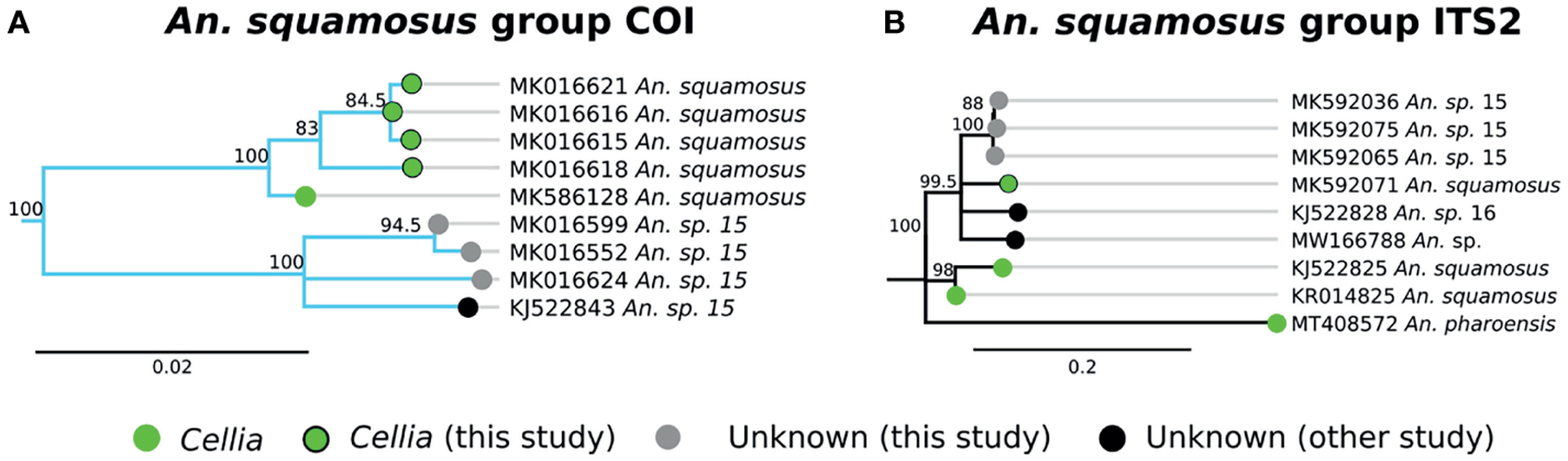
**(A)** Phylogenetic tree based on COI sequences of *An. squamosus* and *An*. sp. *15* group samples. **(B)** Tree based on ITS2 sequences. Trees include both well-defined sequences from this study (green node termini outlined in black) and from NCBI (green node termini without black outline) as well as sequences without a proper species identification (gray from this study and black from NCBI).

A group of 4 specimens clustered with *An. squamosus* COI sequences with high support (Figure 3A, clade E). This clade formed a group with 3 additional specimens that more closely matched a NCBI sequence for “*An*. sp. *15*,” (clade F in Figure 3A) but remained monophyletically clustered with sequence similarity around 94–95% between clades E and F. This is well outside the typical conspecific COI sequence similarity of >98%. All 7 samples were morphologically identified as *An. squamosus* and corresponding ITS2 sequences returned BLAST matches to *An. squamosus*. Given the high relatedness of the *An. squamous* and *An*. sp. *15* COI and ITS2 groupings, it is likely that these belong to the same subgenus, *Cellia*. Variation in ITS2 sequences within *An. squamosus* (82–99% sequence similarities within *An. squamosus* ITS sequences), suggest that there could be higher variation in genetic background within an *An. squamosus* group that could include *An*. sp. *15* and *An*. sp. *16* (Figure 5). Fixed chromosome inversion arrangements have also been identified in *An. squamosus* (personal communication with Maureen Coetzee, University of the Witwatersrand), which supports the likelihood of *An. squamosus* existing as an incompletely described species complex.

### Subgenus Anopheles

**FIGURE 6 |:**
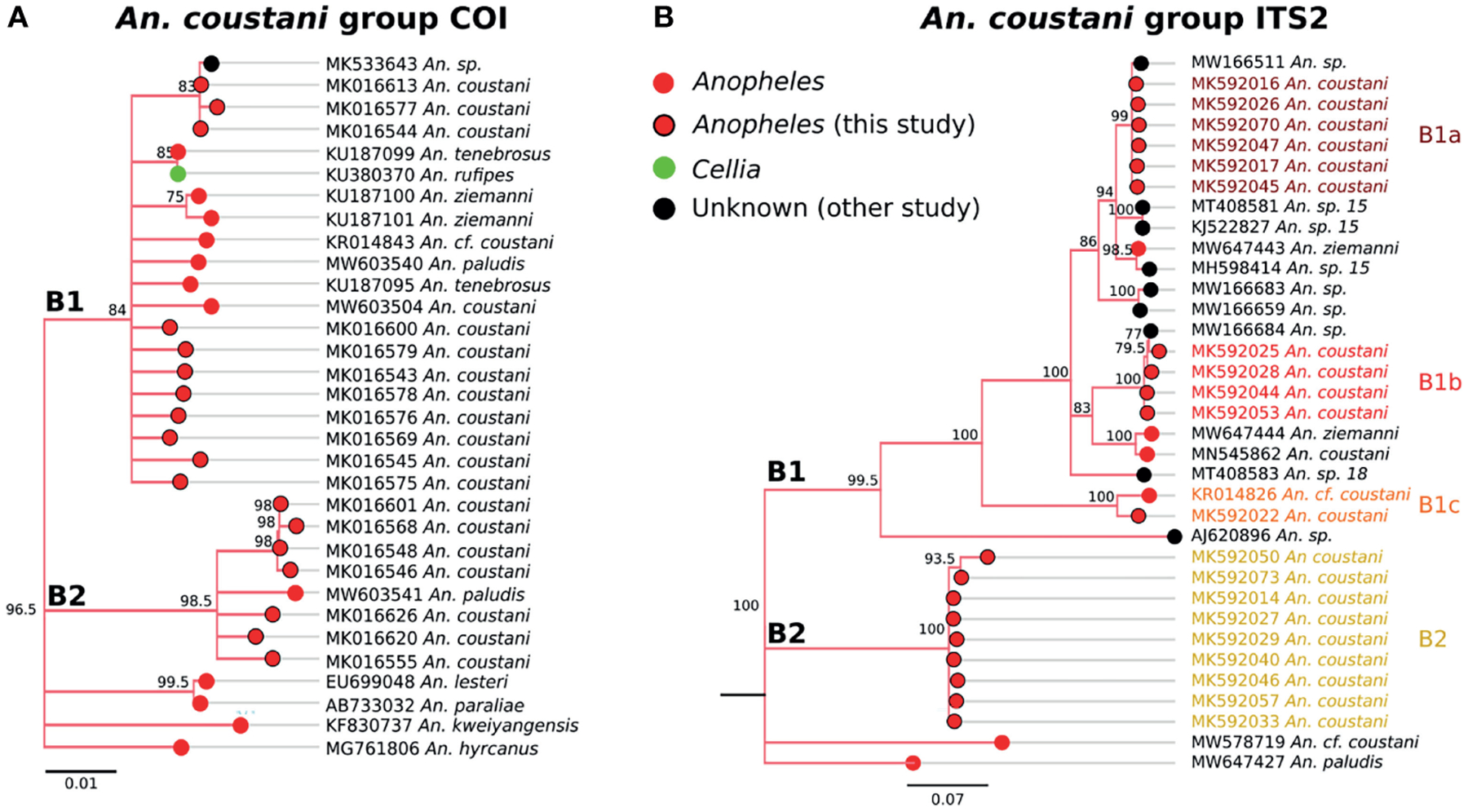
**(A)** Phylogenetic tree based on COI sequences of *An. coustani* group samples. **(B)** Tree based on ITS2 sequences. Trees include both well-defined sequences from this study (red node termini outlined in black) and from NCBI (green or red node termini without black outline), as well as sequences without a proper species identification (black from NCBI).

Two distinct clades were detected within the *coustani* group based on COI sequences (Figure 6). The sequence similarity between the two COI clades (labeled B1 and B2 in Figure 6A) were 96%, which is outside of within-species similarity (>98%) observed in this study. B1 clade includes *An. coustani*, a chromosomal form (cf) of *An. coustani, An. paludis, An. tenebrosus*, and *An. rufipes*. As there was only one *An. rufipes* sequence, a species in the *Cellia* subgenus group (52), inclusion in clade B1 could be due to misclassification. However, if the pattern were repeated with *An. rufipes* samples from other regions, the subgenus designation may need to be revisited. The majority of samples similar to our *An. coustani* group samples belong to the *Anopheles* subgenus. Clade B2 includes one *An. paludis* sample sequenced by another group as well as 7 unique sequences generated from our Zambian samples (Figure 6A).

Genetic relationships among species within clade B1 appear to be complicated and warrant further genetic studies for clarification. Our *An. coustani* samples roughly divided into four groups with one belonging to clade B2 that is distantly related to clade B1 (~58% ITS2 sequence similarity with clade B1). Two closely related ITS2 clades (namely B1a and B1b with 84–85% sequence similarity) and one more distantly related ITS2 clade (B1c with 70% sequence similarity to B1a and B1b) were detected within the B1 clade. Other clades that were not represented in our Zambian samples including *An*. sp. 15 and 18, also grouped with clade B1.

### Other Groups

**FIGURE 7 |:**
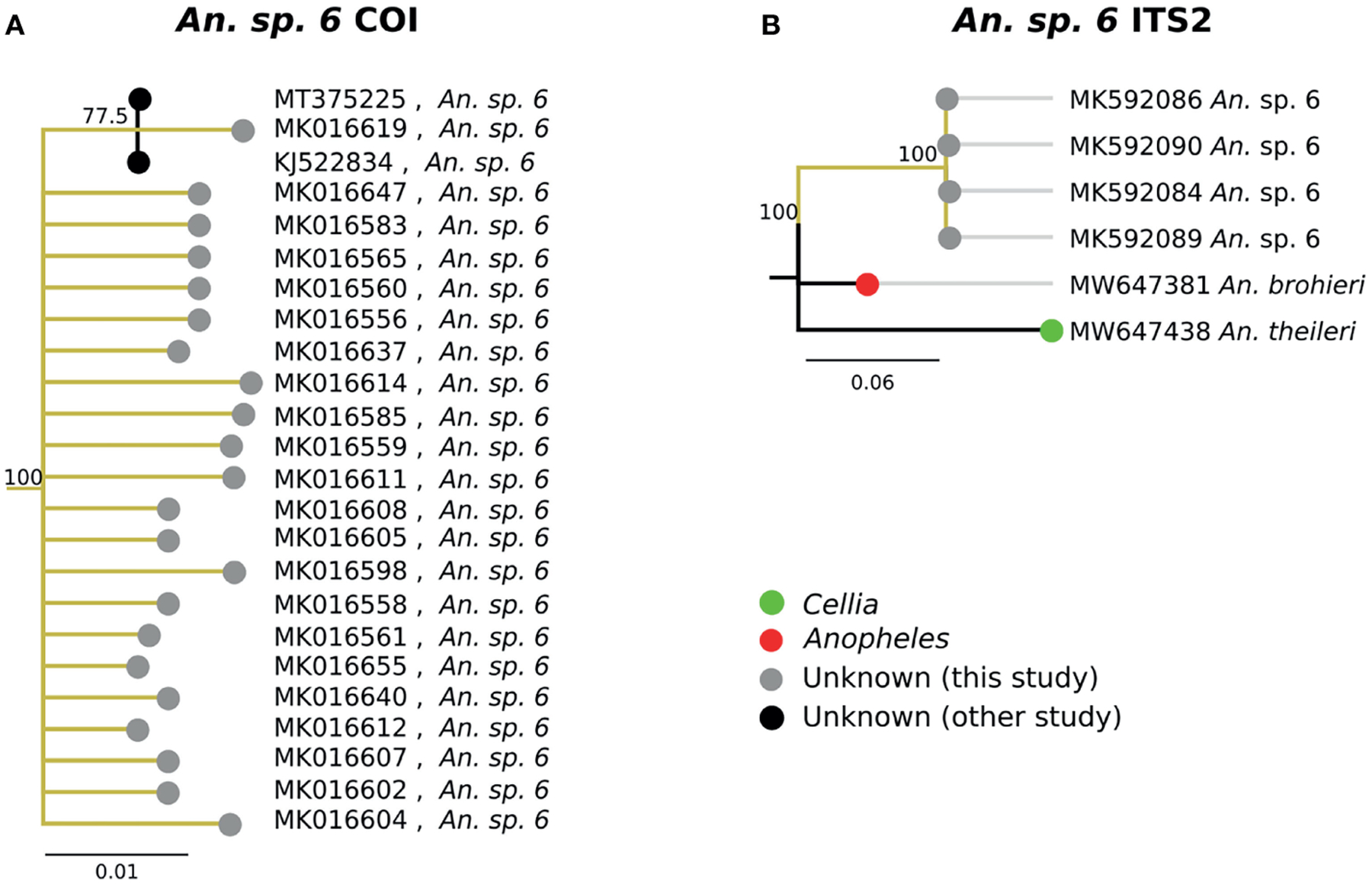
**(A)** Phylogenetic tree based on COI sequences of *An*. sp. *6* samples. **(B)** Tree based on ITS2 sequences. Trees include both well-defined sequences from NCBI (green or red node termini without black outline) as well as sequences without a proper species identification (gray from this study and black from NCBI).

The second-most abundant group collected in the study (31/747, 4.1%) fell within a single highly-supported clade C (Figure 3A). Due to the inclusion in this clade of the sequence of “*An*. sp. *6*” from NCBI, these samples have been classified as *An*. sp. *6*. The *An*. sp. *6* from other studies (MT375225 and KJ522834 in Figure 7A) were from Kenya. The morphological identifications for *An*. sp. *6* specimens were inconsistent and varied: 21/31 (67.7%) were identified as *An. funestus* s.s., 7/31 (22.6%) were identified as *An. gambiae*, and 3/31 (9.68%) were morphologically unclassified. Due to this variability across morphologically disparate groups, the morphology may be best defined as indeterminant. ITS2 sequence similarity between *An*. sp. *6* and *An. theileri* was ~76% (Figure 7B).

**FIGURE 8 |:**
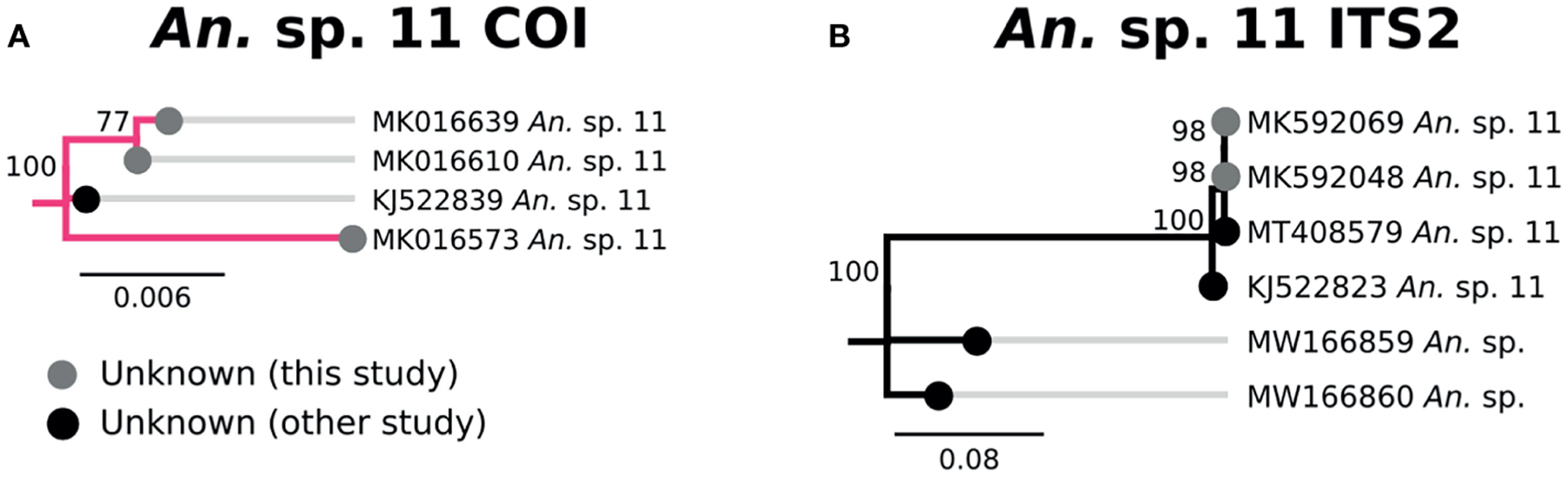
**(A)** Phylogenetic tree based on COI sequences of *An*. sp. *11* samples. **(B)** Tree based on ITS2 sequences. Trees include sequences without a proper species identification (gray from this study and black from NCBI).

Three specimens clustered tightly with *An*. sp. *11* based on COI and ITS2 sequences, and were therefore classified as such (Figure 8). All three were morphologically identified as *An. squamosus*, though neither COI nor ITS2 sequences matched *An. squamosus*. There was no named species that were similar to the *An*. sp. *11* clade. The closest ITS2 sequence matches were from unknown *Anopheles* species from Zambia (53) with 67–68% sequence similarity (Figure 8).

**FIGURE 9 |:**
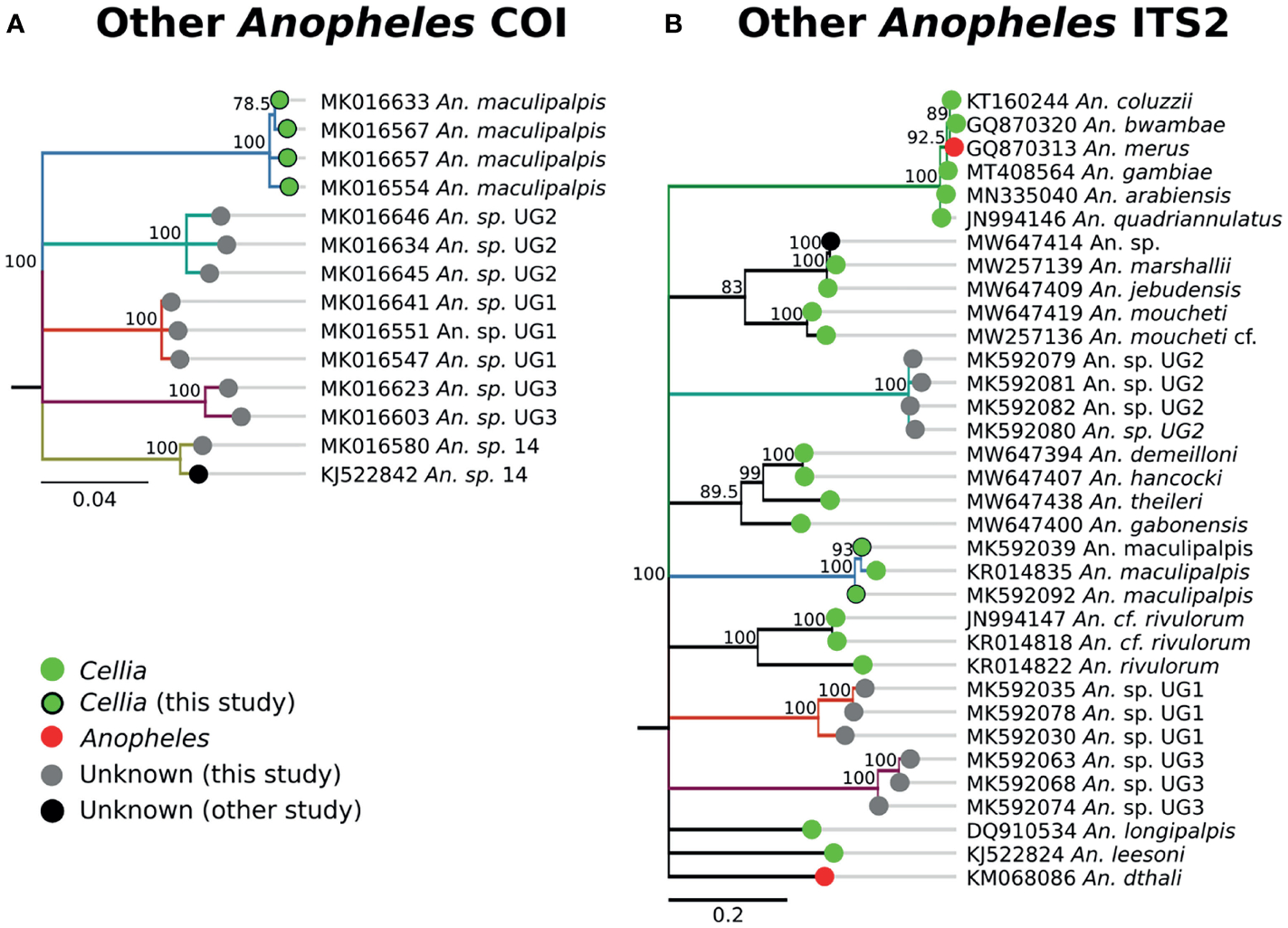
**(A)** Phylogenetic tree based on COI sequences of other *Anopheles* samples. **(B)** Tree based on ITS2 sequences. Trees include both well-defined sequences from this study (green node termini outlined in black) and from NCBI (green or red node termini without black outline), as well as sequences without a proper species identification (gray from this study and black from NCBI).

The single *An*. sp. *14* specimen was morphologically identified as *An. gambiae*. The COI sequences form its own clade (Figures 1, 9) and we did not find any match with ITS2 sequences (Table 1). Consequently, its subgenus placement remains unclear. Three specimens fell into Unknown Group 1 (UG1), five into Unknown Group 2 (UG2), and five into Unknown Group 3 (UG3) (Figure 9). UG1 samples were morphologically identified as *An. funestus* (n = 2) and unidentified (n = 1). ITS2 sequences for this group did not match any existing NCBI data. UG2 and UG3 had varied morphological identifications. One member of UG2 was morphologically unidentified, 2/5 were identified as *An. brunnipes*, and 2/5 as *An. rhodesiensis*. Two members of UG3 were morphologically unidentified, 2/5 were identified as *An. funestus*, and 1/5 as *An. tchekedii*. Neither UG2 nor UG3 COI clustered with significant support with NCBI entries for *An. funestus* and *An. rhodesiensis*, and no COI sequences for *An. brunnipes* nor *An. tchekedii* were available. BLAST results for ITS2 sequences from both groups returned only poor matches to existing sequences in NCBI. Based on our blast results, we can exclude *An. marshallii, An. jebudensis, An. moucheti, An. demeilloni, An. hancocki, An. theileri, An. rivulorum, An. longipalpis, An. leesoni*, and *An. dthali* (Figure 9).

## DISCUSSION

We examined anopheline COI and ITS2 sequences of outdoor foraging mosquitoes to characterize understudied anopheline species and potential malaria vectors in Nchelenge District, Zambia. The collected anophelines included both easily-identified and commonly-recognized major malaria vector species, as well as many species for which species-level identification by morphology was not easily obtained. COI sequences were collected to attain phylogenetic placement of some of these unassigned specimens with well-characterized anopheline species. This allowed for positive identification of some specimens as known species and novel clades for which well-referenced genetic data are not yet available. Determining the species composition of outdoor foraging mosquitoes is critical for guiding malaria surveillance and appropriate interventions as understudied and potential vector species may comprise a large proportion of such collections (34, 37, 54–57).

### Diversity

This collection from outdoor sampling represents a higher diversity of anopheline species than has previously been documented in Nchelenge District, despite extensive sampling in the region spanning almost two decades (38, 39, 58). These studies were focused on indoor collections and predominantly reported *An. funestus* s.s. and *An. gambiae* s.s. The unexpected diversity reported in this study may be explained by several non-mutually exclusive factors. Firstly, this is one of very few studies in the region in which mosquito collections were conducted outdoors, and multiple studies have documented higher species diversity of anophelines outdoors, especially next to livestock. Often routine malaria entomological surveillance is not conducted in such locations (59, 60). Secondly, several consecutive years of IRS combined with insecticide-treated nets (ITNs) targeting *An. funestus* and *An. gambiae*, the most abundant anophelines, may have reduced their populations and other species are now being revealed (42, 61). Finally, the lack of extensive sequencing and rigorous identification of unidentified and assumed non-vector specimens in previous collections may have overlooked existing species (20, 34, 54).

Similar to the *An. gambiae* complex (31), COI was not sufficient to delineate species with in the *An. funestus* group (Figure 4). ITS2 sequences, however, were informative in clustering the clades by known member species. All of our *An. funestus* samples from Nchelenge are *An. funestus* s.s. and no other *funestus* group species were detected in this collection (Figure 4).

*An. squamosus* and its sister species *An. pharoensis* have long been considered secondary vectors of *Plasmodium* to humans (13, 21). Findings from southern Zambia indicate that *An. squamosus* may also serve as a vector of malaria parasites in that region (21). Seven specimens in this study have been tentatively classified as *An. squamosus* based on morphological and molecular data. Three of them match closely (91% ITS2 similarity) with a sequence reported as *An*. sp. *15* (GenBank Accession: KJ522843), which we also tentatively classify as *An. squamosus* on the basis of both morphological identification and molecular analysis (20). *An. squamosus* in southern Zambia has been reported to be comprised of two COI clades, with a strong phylogenetic relationship to *An*. sp. *15* (62), lending support to the hypothesis that these are members of an undescribed species complex. Another sequence reported as *An*. sp. *16* (GenBank Accession: KJ522828) as well as an unknown *Anopheles* sample from Zambia (GenBank Accession: MW166788) are somewhat similar (86–90% ITS2 similarity) to *An. squamosus* ITS2 sequences. Given the close phylogenetic relationship between *An. squamosus* and *An*. sp. *15*, further investigations on the role of these species as alternative vectors of malaria are warranted.

*An. coustani* and the closely-related species *An. ziemanni* and *An. paludis* have likewise increased in notoriety as potentially important malaria vectors in sub-Saharan Africa (55, 63). In some cases, these species have served as major vectors, ranging from Cameroon and the Central Africa Republic to Kenya and Madagascar (64–67). One reason these species have been overlooked for so long is that they have been recognized primarily as exophagic and zoophilic mosquitoes, and so were assumed not to be important in human malaria transmission. However, reports of high degrees of anthropophily in some regions or potentially in some cryptic populations indicate that their vectorial capacity may be much higher (68). The *An. coustani* specimens within this study fell into multiple clades within *An. coustani* group (Figure 6). One subgroup may be *An. coustani* s.s., while the other clades may represent either subpopulations or distinct but highly related species, perhaps within a single species complex. These data are more extensively examined in the context of additional samples from northern Zambia in Ciubotariu et al. (22).

The best match for the second most abundant group of anophelines collected in this study was an NCBI database sequence for “*An*. sp. *6*” (GenBank Accession: KJ522834), which was identified as *An. theileri* group F by another group (20). However, its ITS2 sequences were too divergent from *An. theileri* (~76% ITS2 sequence similarity) to be considered as the *An. theileri* species group. The closest match was *An. brohieri* with 83% ITS2 sequence similarity. *An. brohieri* is in the *Anopheles* subgenus while *An. theileri* is in the *Cellia* subgenus. Available data are not sufficient to conclude that *An*. sp. *6* belongs to the *Cellia* or *Anopheles* subgenus.

*An. maculipalpis*, according to Gillies and De Meillon, is generally a low-abundance species found throughout savannah- and tropical-type environments in Africa and tends to be zoophilic and rest outdoors (69). It has never been implicated as a disease vector of any significant importance. Six samples from our study were identified as *An. maculipalpis* through molecular analysis, although only one of these six was morphologically identified as such. The others were not able to be morphologically identified (N=2) or identified as *An. coustani* (N=3).

Specimens which lack definitive species designations from this study (*An*. sp. *11*, UG1-3) represent anopheline populations which remain unidentified. Proper identification of specimens such as these require not only additional field material for more accurate morphology, but corresponding genetic data from taxonomically-verified specimens. Further studies must be undertaken to properly document these populations to determine if they represent previously undocumented species or are simply species for which we lack genomic information.

### Limitations

The relationships of the anopheline subgenera to one another remain unclear and somewhat contentious (1, 20, 70–74). Studies based on combinations of nuclear and mitochondrial DNA as well as amino acid sequence and morphological characters show that sections/series and even subgenera of anophelines are para-or polyphyletic, i.e., they do not have a single phylogenetic origin. This may be unsurprising, as the original taxonomic classification of anopheline mosquitoes was based largely on morphological characters. As closely-related anopheline species can be morphologically distinct, and distantly-related species remarkably similar, morphological classification may suffer from some degree of evolutionary inaccuracy.

There have been relatively few molecular phylogenetic studies of anopheline mosquitoes at a broad geographic scale. One study used full mitochondrial genomes to analyze the phylogenetics of *Anophelinae* below the genus level (1). Even with much more extensive sequence data than was used in this study, there was low support when using nucleotide data. We attempted to use corresponding amino acid translations for distantly related species, but it did not add discriminatory value to our analyses.

Although the COI BOL is among the most common targets used for phylogenetic analysis in this group of organisms, its utility is likely limited to comparing relatively closely related species as evident by a recent study of the *An. gambiae* complex (31) and *An. funestus* member species relationships illustrated in this study (Figure 4A). Reports have been mixed with regard to the useful phylogenetic signal in COI for comparing subgenera within *Anopheles* (35, 75). To more accurately place ambiguous groups from this study, alternative sequences or multiple targets would be helpful. For instance, ND5 from the mitochondrial genome, along with D2 from the nuclear genome, have been successfully used to resolve relationships at the subgenus level (75, 76). In addition, ITS2 is a very common locus that might be a useful addition and validation of COI-based phylogenetics, although it is difficult to align across taxa and therefore may lead to inaccurate phylogenetic reconstructions.

Morphological misidentification remains a problem, even for experienced investigators (Table 1), particularly when specimens are damaged as commonly occurs during trapping and processing of samples. Misidentification of anophelines for *An. gambiae* specimens in this small sample set was common with 39% (9/23) of specimens morphologically identified as *An. gambiae* being molecularly identified as something else. Comparatively, only 6.2% of morphologically identified *An. funestus* were misidentified. A high proportion of the remaining specimens were morphologically mis- or unidentified, which is likely due to inexperience with identification of relatively rarely observed species of anophelines, as well as damage to specimens in the field or during collection. More extensive documentation of species, including verified voucher specimens for comparison and genetic sequence from such specimens, would be of great benefit to malaria researchers and vector biologists.

### Conclusions

By going beyond standard PCR assays for speciation of samples and conducting phylogenetic analysis, this study was able to show an unprecedented diversity of anophelines in Nchelenge District, northern Zambia. Several of these anophelines represent species known to be or emerging as important vectors for malaria transmission in other regions of Africa. At such low numbers in this collection, it is impossible to estimate their contribution to transmission in Nchelenge District and long-term studies of outdoor anophelines spanning larger parts of the district and region are required to further determine their role and distribution. Other specimens in this study remain unverified and represent either unnamed species or named species which have yet to be genetically characterized. Future taxonomic efforts are clearly needed to link anopheline morphology to genomic data.

## Supplementary Material

Supplementary table **Supplementary Table S1 |** Genbank accession numbers of COI and ITS2 sequences used in our analysis.

## Figures and Tables

**TABLE 1 |:** Phylogenetic groups confirmed through PCR and sequencing (N=747).

Clade	N	Morphological ID	COI	Best matched species based on COI	COI % sequence identity	ITS2	Best matched species based on ITS2	ITS2% sequence identity	Consensus species ID	Subgenus
A	644	*An. funestus*	26	*An. funestus*	>99%	1	*An. funestus*	>99%	*An. funestus*	*Cellia*
B	28	*An. coustani*	28	*An. coustani* group	>95%	24	*An. coustani* *An. cf. coustani*	>72%	*An. coustani* group	*Anopheles*
C	31	*An. funestus or An. gambiae or undetermined*	29	*An*. sp. *6*	>99%	11	*An. brohieri*	93.1%	*An*. sp. *6*	Unknown
D	14	*An. gambiae*	3	*An. gambiae s.l*.	>99%	2	*An. gambiae s.l*.	>99%	*An. gambiae*	*Cellia*
E	4	*An. squamosus*	4	*An. squamosus*	>99%	1	*An. squamosus*	92.4%		*Cellia*
F	3	*An. squamosus*	3	*An*. sp. *15*	>98%	3	*An*. sp. *16*	92.9–93.3%		*Cellia*
G	6	*An. coustani*	5	*An. maculipalpis*	>99%	2	*An. maculipalpis*		*An. maculipalpis*	*Anopheles*
H	3	*An. squamosus*	3	*An*. sp. *11*	>99%	2	*An*. sp. *11*	>99%	*An*. sp. *11*	*Cellia*
I	5	*An. funestus*	5	*An*. sp. *6* *An*. sp. *14*	~95.5%	3	*An. demeilloni*	70.8%	Unknown *An*. (UG1)	Unknown
J	5	*An. brunnipes* *An. rhodesiensis*	5	*A-L*	92–93%	4	*An. marshalli*	77%	Unknown *An*. (UG2)	Unknown
K	1	*An. gambiae*	1	*An*. sp. *14*	98.6%	1	*An*. sp. *14*	NA	*An*. sp. *14*	*Cellia*
L	3	*An. funestus* *An. tchekedii*	3	*An*. sp. *6*	~93%	3	*An. demeilloni* *An. hancocki*	76.7–76.8%	Unknown *An*. (UG3)	*Cellia*

Species for which the molecular ID differed from the morphological ID are marked in red font. NA, not available.

## Data Availability

The datasets presented in this study can be found in online repositories. The names of the repository/repositories and accession number(s) can be found in the article/Supplementary Material.
